# Clinical profiles, comorbidities, and treatment outcomes of stroke in the medical ward of Dessie comprehensive specialized hospital, Northeast Ethiopia; a retrospective study

**DOI:** 10.1186/s12883-022-02916-7

**Published:** 2022-11-01

**Authors:** Hussen Abdu, Fentaw Tadese, Girma Seyoum

**Affiliations:** 1 Department of Anatomy, School of Medicine, College of Health Sciences, Wollo University, Dessie, Ethiopia; 2 Department of Epidemiology and Biostatistics, School of Public Health, College of Medicine and Health Sciences, Wollo University, Dessie, Ethiopia; 3 Department of Anatomy, College of Health Sciences, Addis Ababa University, Addis Ababa, Ethiopia

**Keywords:** Clinical profile, Comorbidity, Mortality, Stroke, Treatment outcome

## Abstract

**Background:**

Undoubtedly, stroke is expanding as a global public health issue. Stroke-related deaths are attributable to modifiable risk factors. A demographic shift in Ethiopia increased the prevalence of stroke risk factors. Furthermore, there is limited relevant information available about stroke. Therefore, the current study sought to evaluate the clinical profiles, comorbidities, and treatment outcomes of stroke in the medical ward of Dessie comprehensive specialized hospital.

**Methods:**

A retrospective cross-sectional study design was employed among stroke patients. The study included medical records with complete patient information and a stroke diagnosis that had been verified using imaging techniques. Using simple random sampling, 344 medical records were selected, 312 of which met the requirements for inclusion. The frequency and percentage of sociodemographic characteristics and other variables were described using descriptive statistics.

**Results:**

The patients were 59.2 ± 14.6 years old on average. About 14.7% of the study participants were chat chewers. Of stroke victims, about 52.2% had sensory loss and limb weakness. Nearly 44.9% of the patients had hemiplegia or hemiparesis when they were first seen, and 25.3% were unconscious. Hypertension (63.1%), atrial fibrillation (15.1%), and structural heart diseases (12.5%) were the frequently seen co-morbidities in stroke patients. About 35.8% of the patients had fully recovered and were released go from the hospital without suffering any repercussions. However, hospital deaths from stroke accounted for 21.8% of cases. Stroke fatalities usually involved hypertension, atrial fibrillation, and structural heart disorders.

**Conclusion:**

Sensory deficits, limb weakness, and mentation loss were all common clinical presentations in stroke patients. In particular, hypertension, atrial fibrillation, and structural heart diseases were commonly seen as comorbidities in stroke patients. Stroke mortality was high in the hospital. Thus, establishing promotive, preventive, curative, and rehabilitative strategies is indispensable.

## Introduction

A stroke, also known as a cerebrovascular accident, is a localized or widespread impairment of cerebral function brought on by blockage or rupture of blood vessels that supply the brain, with symptoms lasting 24 h or longer or fatalities with no other obvious explanation other than that of vascular origin [1, 2].

Without a blood supply, brain cells begin to deteriorate and eventually die, resulting in permanent brain damage [3–5]. Some sick individuals luckily recover entirely following strokes. However, more than two thirds of survivors will have some sort of lasting disability [6, 7].

Stroke is among the leading causes of morbidity and mortality worldwide [8]. It is a collection of devastating and debilitating diseases. Stroke is an issue that drastically lowers a person’s overall quality of life. In 2017, it was the third most common cause of death globally, with a high frequency of around 15 million specific cases annually. It is responsible for 10% or so of all deaths [9]. 70% of strokes worldwide and 87% of stroke-related deaths and disability-adjusted life years occur in low- and middle-income countries [10].

Globally, modifiable risk factors are responsible for 90% of cardiovascular illnesses, including stroke and myocardial infarction [11]. In 2017, modifiable risk factors were responsible for 5.2 million stroke-related deaths and 116.3 million stroke-related disability-adjusted life years lost globally [12].

Stroke, which accounts for 24% of all neurological admissions in Ethiopia, continues to be the most common neurological illness among patients who are hospitalized [13–15]. Currently, stroke deaths in Ethiopia account for 4.71% of all deaths. Ethiopia is ranked 107th in the world for age-adjusted mortality rates, with 71.94 deaths per 100,000 people [16].

A demographic and epidemiologic shift in Ethiopia raised the prevalence of stroke risk factors. Furthermore, there is a lack of information on the clinical characteristics, risk factors, and prognoses of stroke therapy in Ethiopia [15] and at Dessie Comprehensive Specialized Hospital (DCSH) too. Additionally, up-to-date information on stroke is essential for designing, implementing, and assessing stroke prevention, acute care, and rehabilitation programs at home for people with disabilities. We thus made an effort to evaluate the clinical profiles, comorbidities, and treatment outcomes of stroke among patients admitted to the medical ward of DCSH during the study period.

## Materials and methods

We employed analogous materials and methodologies, and the data in this analysis was a component of our earlier work. As a result, our earlier paper and the study participants’ characteristics in this study are related [17].

### Study Design, setting, and period

An institution-based cross-sectional study was carried out from January 2016 to December 2019 among stroke patients hospitalized in the medical ward of DCSH. This public hospital is located 401 km from Addis Ababa the capital city of Ethiopia, in the eastern parts of the Amhara regional state, in the Dessie municipal government. It is one of the oldest hospitals in the Amhara regional state and serves over 10 million people.

### Study Population

The study population was all systematically selected stroke patients admitted in the medical Ward of DCSH during the study period.

### Eligibility criteria

Stroke cases confirmed using computerized tomography scan or magnetic resonance imaging and admitted to the medical ward of DCSH during the study period were included in the study. Medical records with incomplete required information were excluded from the study.

### Sample size and Sampling Procedure

The sample size was determined using Epi Info 7 with the help of single population proportion formula, taking into account the expected mortality proportion amongst stroke patients who were hospitalized at 13%, a 95% confidence level, and a 3% margin of error [14]. The total expected patient flow was 1200. Based on this assumption, the sample size was 344. Using simple random sampling, 344 medical records were selected from a total of 1371 hospitalized stroke patients, and 312 of those records met the inclusion criteria and included in the study.

### Data Abstraction Tools and Procedure

The necessary data were carefully abstracted by trained medical interns using a professionally prepared checklist. The checklist was prepared by reviewing selected medical records.

### Dependent variables

Stroke leads to a range of unfavourable functional outcomes. Improving the post-stroke disability and functional independence has been the core rehabilitation content among stroke patients [18]. In-hospital outcomes of stroke patients represent the dependent variables and evaluated based on the information filled on the discharge forms [19].

### Independent variables

The independent variables represent the socio-demographic characteristics of the patients like sex, age, and residence. Moreover, co-morbidities like hypertension, diabetes, atrial fibrillation, structural heart diseases, previous history of stroke, family history of stroke, obesity, headache, and HIV infections, were independent variables. Also, behavioral characteristics like smoking, alcohol intake, and chat chewing were justly considered the independent variables.

## *Operational definitions*

- **Alcohol intake**: Any amount of alcohol consumption [20].
- **Stroke**: defined as “rapidly developing clinical signs of focal (global) disturbance of cerebral function lasting longer than 24 hours unless interrupted by death with no apparent cause other than that of vascular origin” confirmed with CT scan/MRI [1, 2].
- **Disability Adjusted Lived Years (DALYs)**: Years in which the stroke patients lived with the physical disabilities and functional impairments caused by stroke [21].
- **Stroke patients’ outcomes** [17]
- **Complete resolution from stroke**: is when an individual stroke patient is properly discharged with better health and relieved from clinical complaints as well as none of the potential complications are sufficiently developed.
- **Discharged with a neurologic deficit (DWND)**: a stroke patient with improved signs and symptoms but discharged with stroke complications like physical impairment, cognitive impairment, and communication impairment.
- **Death**: when a loss of life occurs because of stroke and its complications.
- **Discharged against medical advice (DAMA)**: - is when stroke patients refuse all the medical advice despite their health status and treatment outcomes.
- **Glasgow coma scale (GCS)**: it helps to measure the level of consciousness (13)
- Good GCS (13–15): mild brain injury (alert).
- Moderate GCS (9–12): moderate brain injury (drowsy).
- Poor GCS (≤ 8): severe brain injury (unconscious).
- **Neurological manifestations** .[22, 23]
- **Loss of consciousness**: inability to remain awake, aware, and oriented to place, people and time.
- **Aphasia**: is a language disorder that affects the ability to communicate. It’s most often caused in the left side of the brain that control speech and language.
- **Urinary incontinence**: refers to inability of bladder control which is common among stroke survivors.
- **Hemiplegia/Hemiparesis**: is weakness or the inability to move on one side of the body, making it hard to perform everyday activities like eating or dressing.
- ***Grasp reflex***: is an involuntary flexion-adduction movement involving the hands and digits
- **Facial palsy**: refers to weakness of the facial muscles, mainly resulting from temporary or permanent damage to the facial nerve
- **Dysarthria**: difficulty speaking caused by brain damage, which results in an inability to control the muscles used in speech.
- **Quadriplegia**: refers to paralysis from the neck down, including the trunk, legs and arms.
- **Hemianopia**: is when an individual lose sight in half of his/her visual field.
- **Diplopia**: is seeing two images of a single object when looking at it.
- **Ataxia**: is a term for a group of disorders that affect co-ordination, balance and speech.
- **Convulsion**: is an episode in which the person experience rigidity and uncontrolled muscle spasms along with altered consciousness.

### Data Analysis

The extracted data were reviewed, cleaned, and entered into SPSS version 24.0 software for analysis. Descriptive statistics were used to characterize the frequency and percentage of sociodemographic factors and other variables. The results were presented in the form of texts, tables, and figures.

## Results

### Socio-demographic characteristics of the study subjects

In the study, women made up about 51.9% of the study subjects. The average of the study patients was 59.2 ± 14.6 years. The majority of participants (41.7%) were between the ages of 65 and 84. In addition, 65.7% of the participants were from rural areas, making up two-thirds of the study participants. Additionally, about 14.7%, 12.2%, and 10.3% of the study participants were chat chewers, alcohol consumers and cigarette smokers, respectively. Furthermore, about 5.4% of the study participants had previous history of stroke (Table 1).

**Table 1:** Socio-demographic characteristics of stroke patients admitted at the medical ward of Dessie comprehensive specialized hospital, January 2016-December 2019

Variables	Categories		Frequency	%
Sex	Male		150	48.1
	Female		162	51.9
Age	< 25		10	3.2
	25–44		36	11.5
	45–64		120	38.5
	65–84		130	41.7
	≥ 85		16	5.1
Residence	Rural		205	65.7
	Urban		107	34.3
Religion	Muslim		172	55.1
	Orthodox		115	36.9
	Protestant		20	6.4
	Catholic		5	1.6
Educational status	No formal Education		184	59.0
	Primary school		36	11.5
	Secondary School		24	7.7
	College and above		16	5.1
	Not specified		52	16.7
Marital status	Single		5	1.6
	Married		234	75.0
	Divorced		28	9.0
	Widowed		16	5.1
	Not specified		29	9.3
Life style history	Alcohol intake	Yes	32	10.3
		No	280	89.7
	Cigarette smoking	Yes	38	12.2
		No	274	87.8
	Chat chewing	Yes	46	14.7
		No	266	85.3
	Obesity	Yes	16	5.1
		No	296	94.9
History of stroke	Family history	Yes	15	4.8
		No	297	95.2
	Previous history	Yes	17	5.4
		No	295	94.6

### Clinical presentations

About 52.2% of stroke patients lost feeling in certain areas of their bodies and experienced weakness when they arrived at the hospital. Approximately 44.9% of patients had hemiplegia or hemiparesis and 25.3% of the patients were unconscious when they first arrived. At the time of admission, the patients had a mean Glasgow coma scale of 11.18 ± 3.30 (mean ± SD), ranging from four to fifteen. The patients’ average systolic and diastolic blood pressures were 154.2 ± 31.4 mmHg and 94.5 ± 18.9 mmHg, respectively (Table 2).

**Table 2:** Clinical profiles of stroke patients admitted at the medical ward of Dessie comprehensive specialized hospital, January 2016-December 2019

Clinical Presentations	Frequency	%
Loss of consciousness	79	25.3
Weakness and Sensory loss	163	52.2
Aphasia	63	20.2
Urinary incontinence	67	21.5
Hemiplegia/Hemiparesis	140	44.9
Grasp reflex	5	1.6
Facial palsy	28	9.0
Dysarthria	7	2.2
Quadriplegia	4	1.3
Headache	11	3.5
Hemianopsia	7	2.2
Diplopia	9	2.9
Visual perceptual deficits	13	4.2
Ataxia and incoordination	19	6.1
Vomiting	23	7.4
Convulsions	6	1.9
SBP (mean ± SD)	154.2 ± 31.4 mmHg	
DBP (mean ± SD)	94.5 ± 18.9 mmHg	
GCS (mean ± SD)	11.18 3.30	

### Comorbidities on stroke patients

In terms of comorbidities, hypertension was the most prevalent condition seen in 63.1% of the study participants. The study participants also had atrial fibrillation (15.1%), structural heart diseases (12.5%), and both hypertension and diabetes (9.6%) (Table 3).

**Table 3:** Prevalence of comorbidities in stroke patients at the medical ward of Dessie comprehensive specialized hospital, January 2016-December 2019

Comorbidities	Categories	Frequency	%
Hypertension	Yes	197	63.1
	No	115	36.9
Diabetes	Yes	24	7.7
	No	288	92.3
Atrial fibrillation	Yes	47	15.1
	No	265	84.9
Structural Heart Diseases	Yes	39	12.5
	No	273	87.5
Hypertension & Diabetes	Yes	30	9.6
	No	282	90.4
HIV	Yes	17	5.4
	No	295	94.6
Headache/migraine	Yes	21	6.7
	No	291	93.3

### Treatment outcomes of stroke

**Fig. 1:**
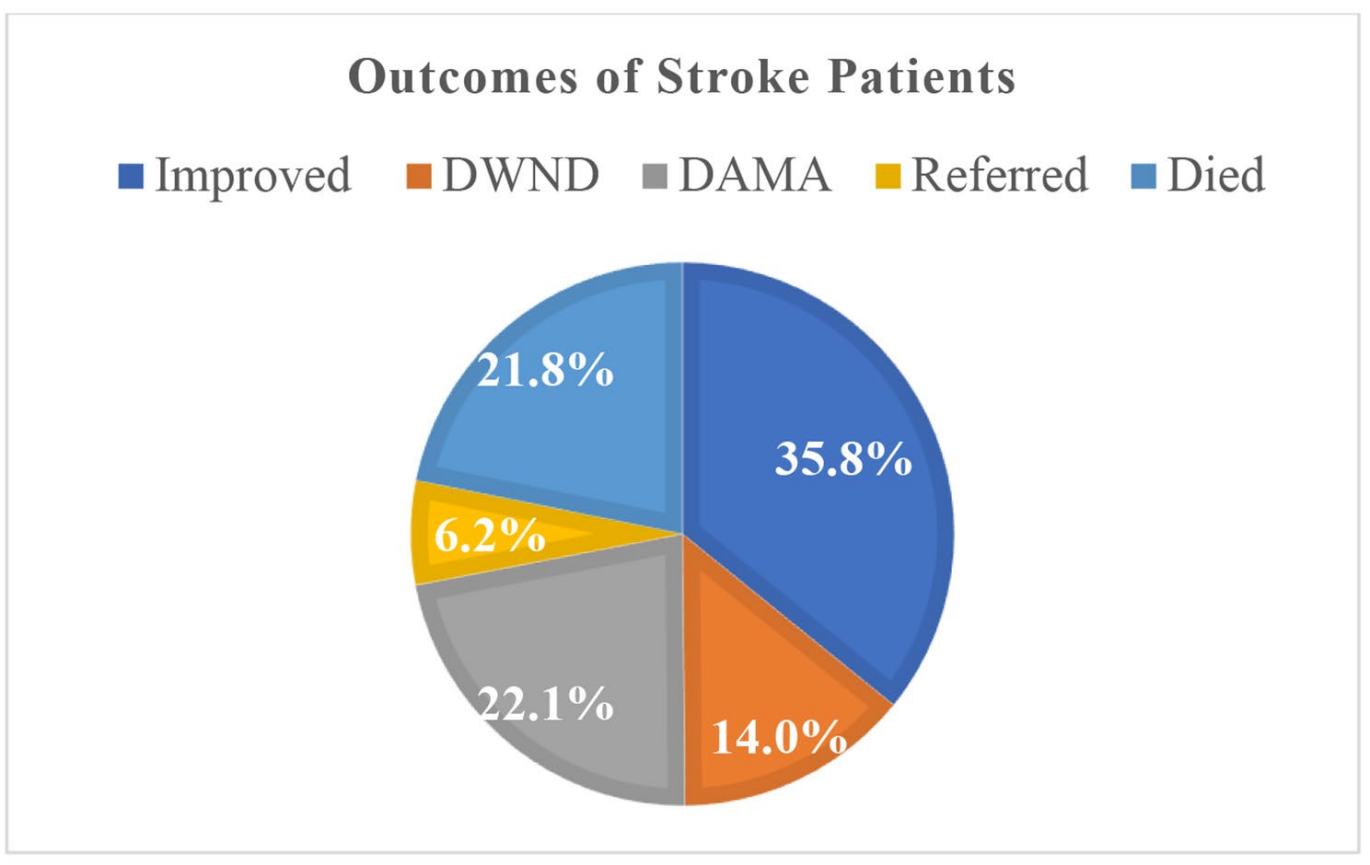
Treatment outcomes for stroke patients admitted at the medical ward of Dessie comprehensive specialized hospital, January 2016-December 2019; DWND-Discharged with Neurologic Deficit, DAMA-Discharged against Medical Advice

Five patients’ outcome statuses were not available in their medical records. So, we looked at the results of 307 individuals. Approximately 35.8% of the patients had their problems totally cured and were discharged from the hospital without experiencing any complications. About 22.1% of patients left the hospital against the advice of the medical staff. In addition, about 21.8% of the patients with strokes at the DCSH medical ward died in the hospital (Fig. 1).

**Fig. 2:**
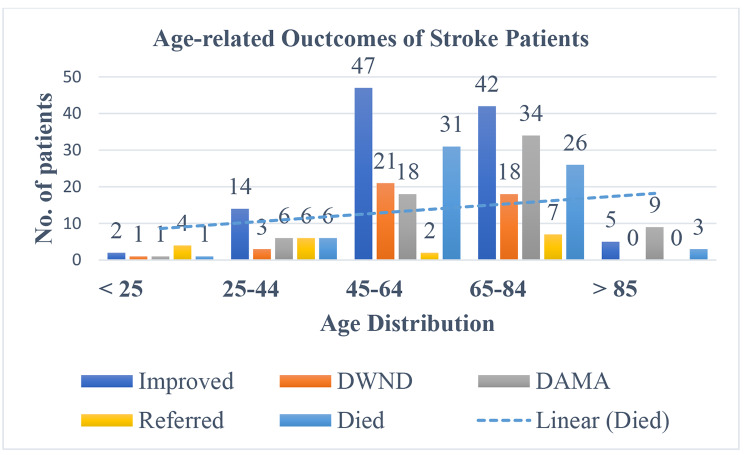
Age-related outcomes of stroke patients admitted at the medical ward of Dessie comprehensive specialized hospital, January 2016-December 2019. DWND- Discharged with Neurologic Deficit, DAMA- Discharged against Medical Advice

According to Fig. 2, out of 67 deaths that were reported, 31 happened in the age category of 45 to 64 years, followed by 65 to 84 years, where 26 deaths were reported. From this result, the tendency for mortality from strokes rose with age. Furthermore, in the current study, 14.3% of stroke-related deaths had hypertension, 4.9% had AF and SHD (Table 4).

**Table 4:** The proportion of factors for stroke-related mortality at the medical ward of Dessie comprehensive specialized hospital, January 2016–December 2019

Risk factors	Categories	Frequency of deaths (67)	Percentage (21.8%)
Types of strokes	IS	42	13.7
	HS	25	8.1
Hypertension	Yes	44	14.3
	No	23	7.5
Diabetes mellites	Yes	7	2.3
	No	60	19.5
Diabetes and Hypertension	Yes	6	2.0
	No	61	19.8
Previous history of stroke	Yes	9	2.9
	No	58	18.9
Atrial fibrillation	Yes	15	4.9
	No	52	16.9
Smoking	Yes	10	3.3
	No	57	18.5
Alcohol intake	Yes	8	2.6
	No	59	19.2
Family history of stroke	Yes	5	1.6
	No	62	20.2
Structural heart diseases	Yes	15	4.9
	No	52	16.9
HIV	Yes	6	2.0
	No	61	19.8
Chat chewing	Yes	13	4.2
	No	54	17.6
Obesity	Yes	6	2.0
	No	61	19.8
Headache	Yes	5	1.6
	No	62	20.2

HIV- Human Immune Virus, HS- Hemorrhagic Stroke, IS- Ischemic Stroke

## Discussion

Stroke and other chronic, non-communicable illnesses are becoming major global public health issues. Stroke remains to be the most prevalent neurological illness among patients admitted to hospitals in Ethiopia. It accounts for 24% of all neurological hospitalizations and is linked to high morbidity and mortality [13, 14]. This study examined the clinical manifestations, risk factors, and outcomes of stroke therapy at the study hospital.

In the current study, women were affected by stroke at a relatively higher rate than men. This result agrees with other earlier studies carried out in Ethiopia [14, 24]. This might be caused by problems specific to women, such as physiologic changes and diseases associated with pregnancy, risk factors associated with menstruation, or the use of contraceptives during a woman’s reproductive life. However, this finding contradicts various studies from both developing and developed countries that found more men than women were afflicted by stroke [13, 19, 25–28].

The age distribution of stroke in our research was in line with those of previous comparable studies carried out in various parts of Ethiopia [14, 19, 29]. However, this result was different from a study conducted at Tikur Anbesa Specialized Hospital in Addis Ababa, where the majority of stroke patients were under the age of 34 [24].

The patients in our study had a mean age of 59.2 ± 14.6 years. This is consistent with earlier hospital-based studies done in Ethiopia and other SSA nations, which found that the average age of stroke patients was between 55 and 65 years old [13, 14, 19, 29–33]. However, the median age of the patients in this study was lower than that reported in prior studies done at the Gondar University Hospital and in western nations, which both declared a median age of 68 years [14] and 73 years [34], respectively. This high frequency of stroke in older people may be brought on by the existence of age-related cardiovascular comorbidities, such as diabetes and hypertension, which are also possible risk factors. Stroke tends to happen a few years earlier in developing nations like Ethiopia than in western nations. This discrepancy may be a possible sign that hospital-based studies are susceptible to selection bias and have subpar risk factor control measures. In order to ascertain the age distribution of stroke in both our research regions and the nation as a whole, community-based studies are necessary.

More than half of stroke patients had sensory and motor symptoms when they were first admitted. Likewise, hemiplegia/hemiparesis was listed as the most typical clinical presentation in stroke patients [14, 24, 26, 35]. This striking similarity might be explained by the use of a comparable data collecting technique, in which motor symptoms and other clinical characteristics were gathered simultaneously. About 25.3% of the study subjects were unconscious or lost their mentation while being admitted. Incontinence, expressive aphasia, facial palsy, vomiting, ataxia, unsteadiness, anomalies in visual perception, and headache were among the most common clinical symptoms. In contrast to earlier studies [14, 26, 28, 36], the frequencies of these clinical manifestations were lower in our study. The average clinical presentation for the patient was 4, which was less than the study from Jimma University Hospital, which reported an average clinical presentation of 6 [28] and comparable to a study from India, which showed 3–4 clinical symptoms upon admission [37].

Elevated levels of both systolic and diastolic blood pressure, which indicated hypertension, were present in the majority of the study participants. This is consistent with a number of other research that showed elevated systolic and diastolic blood pressure values in stroke patients [14, 15, 28, 29, 31, 38–40]. This agreement may be the result of similarities in the evaluation methods used to measure blood pressure or because hypertension is regarded as a common risk factor for stroke in the general population of the world. The average GCS score for the patients was 11.18 with a standard deviation of 3.30, indicating a moderate level of brain damage [41]. This is in agreement with another earlier study [28].

In the present study, hypertension was the comorbidity with the highest prevalence rate among stroke patients. Similar to this, several studies [14, 15, 28, 29, 31, 38–40] identified hypertension as the most prevalent comorbidity seen in stroke patients. In most studies, however, there were varying numbers of hypertensive patients. Yet, these inconsistencies may have gotten up as a result of the categorization of hypertension based on the values of systolic and diastolic blood pressure that were recorded at the time of hospital arrival with agitated patient conditions or may have been caused by subpar measurement tools and professional skills. Aside from hypertension, atrial fibrillation, and both diabetes and hypertension were important comorbidity of stroke in the current study. We deduced from this that cardiovascular problems, hypertension, and diabetes are all linked to the development of stroke.

The majority of the study participants showed complete resolution and were discharged from the hospital without any obvious complications. This concurs with other institution-based studies conducted in Ethiopia [14, 33, 42]. The percentage of deaths in the current study was high as compared with other previous studies [13, 14, 29]. Yet, it was comparable with a study done at Tikur Anbesa specialized hospital, Addis Ababa, where a 20% of the stroke mortality rate was reported [43] and lower than other studies [19, 44]. These differences could be due to the high rate of patients leaving the hospitals against medical advice when the patient’s condition was worsening. The outcome of the patients was significantly affected by their age. Stroke fatalities usually involved hypertension, atrial fibrillation, and structural heart disorders. These results were also similar to studies conducted at St. Paul’s teaching hospital [19].

## Conclusion and recommendations

Elders were more affected by stroke. Stroke patients frequently presented with sensory impairments, limb weakness, and mentation loss. Patients with stroke frequently have concomitant conditions, such as hypertension, atrial fibrillation, and structural heart diseases. Most stroke victims recovered from their issues. However, still some patients left the hospital with neurologic deficits. In the study hospital, the percentage of stroke-related mortality remained to rise. The tendency for mortality from strokes rose with age. As a result, developing promotional, preventative, curative, and rehabilitative measures is essential.

## Limitations of the study

Getting complete information and avoiding partialities were challenging in the retrospective type of study. The hospital has no standard scales for measuring patient outcomes. Therefore, standard scales were not used to assess patient outcomes.

## Data Availability

The datasets used and/or analysed during the current study are available from the corresponding author on reasonable request.

## Abbreviations

DCSH: Dessie Comprehensive Specialized Hospital
GCS: Glasgow Coma Scale
HIV: Human Immune Virus
SPSS: Statistical Package for Social Sciences
WHO: World Health Organization
